# Genotoxicity of polystyrene and polyethylene micro- and nanoparticles in human lung (A549) and liver (HepG2) cell lines

**DOI:** 10.3389/ftox.2026.1962775

**Published:** 2026-10-21

**Authors:** Emilija Striogaitė, Vaiva Horbačauskaitė, Milda Babonaitė-Aleksiejūnė, Juozas Rimantas Lazutka

**Affiliations:** Department of Botany and Genetics, Institute of Biosciences, Life Sciences Center, Vilnius University, Vilnius, Lithuania

**Keywords:** comet assay, genotoxicity, micro- and nanoplastics, polyethylene, polystyrene

## Abstract

Micro- and nanoplastics (MNPs) are widespread environmental pollutants that have raised concerns regarding their potential impact on human health. Among the most common plastic polymers, polystyrene (PS) and polyethylene (PE) are frequently detected in the environment, though comparative information on their biological effects remains limited. This study investigated the cellular responses to PS and PE MNPs in the A549 and HepG2 cell lines, with particular emphasis on cytotoxicity, primary DNA damage, oxidative stress, oxidative DNA damage, and particle uptake. Cytotoxicity was evaluated through dual acridine orange and ethidium bromide staining, while primary and oxidative DNA damage was measured using alkaline and Fpg-modified comet assays, respectively. Particle uptake and reactive oxygen species (ROS) generation were assessed by flow cytometry. Both PS and PE particles induced modest reductions in cell viability. HepG2 cells exhibited greater susceptibility to primary DNA damage, with PS particles inducing significant damage only in HepG2 cells, whereas PE particles induced significant DNA damage in both cell lines, particularly at higher concentrations. No consistent concentration-dependent increase in ROS levels or oxidative DNA damage was observed following exposure to PS and PE particles, despite statistically significant changes in DCF fluorescence under selected exposure conditions. Both particle types altered side-scattering (SSC) signal intensity in A549 and HepG2 cells, with a more pronounced signal observed in A549 cells. MNP-induced genotoxic responses varied according to polymer type and cellular model and were not consistently associated with ROS-mediated oxidative DNA damage.

## 1 Introduction

The rapid increase in global plastic production has created a significant environmental challenge. Since the 1950s, plastic production has increased more than 200-fold (Ritchie et al., 2023). Plastic pollution is an environmental issue that highlights the critical importance of effective waste management rather than merely focusing on the volume of plastic produced. According to the Organization for Economic Co-operation and Development’s Global Plastics Outlook, around 20% of the world’s plastic waste is mismanaged (OECD, 2019). Various physical, chemical, and biological processes can fragment larger plastic debris into smaller micro- and nanosized particles, which then interact with biological systems. These particles, particularly polystyrene (PS) and polyethylene (PE), which are among the most prevalent plastic polymers, have been detected in various environmental compartments, raising concerns about their potential impacts on organisms and human health (Cooper and Corcoran, 2010; Duis and Coors, 2016; Rani, 2022; Schmidt et al., 2026).

The biological effects of plastic particles are influenced by properties such as polymer composition, particle size, surface characteristics, and chemical additives (Lu et al., 2016; Nolte et al., 2017; Diebold et al., 2022). These characteristics can influence particle stability and cellular uptake, thereby contributing to oxidative stress, inflammation, and cellular toxicity (Lu et al., 2016; Nolte et al., 2017; Lin et al., 2025).

Polystyrene nanoparticles have been shown to be taken up by human lung cells in a time-, dose-, and particle-size-dependent manner, with increased uptake observed at higher concentrations and after longer exposure periods (Liu et al., 2022). Following cellular uptake, polystyrene and polyethylene particles may induce mitochondrial dysfunction and oxidative stress, which can subsequently contribute to inflammatory responses and apoptosis (Liu et al., 2021; Dal Yöntem and Ahbab, 2024; Huovinen et al., 2026). The generation of ROS is considered an important mechanism underlying these responses, as excessive ROS production may disrupt cellular homeostasis and contribute to DNA damage. Smaller PS particles have been associated with greater increases in oxidative DNA damage, although the relationship between particle accumulation and genotoxic responses may not be linear (Halimu et al., 2022; Guimarães et al., 2023). Polyethylene microparticles have been reported to induce not only DNA damage but also chromosomal damage, as evidenced by a significant increase in the frequency of micronuclei (Çobanoğlu et al., 2021; Abdel-Zaher et al., 2027). Genotoxicity is particularly significant, as it can lead to long-term biological consequences, including mutations and impaired cellular function.

However, the comparative genotoxic effects of different plastic polymer particles remain insufficiently understood. Differences in polymer structure and chemical composition may influence their interactions with cells and their capacity to generate oxidative stress or other processes that lead to DNA damage (Płuciennik et al., 2024). As cellular susceptibility to MNPs may vary between cell types (Płuciennik et al., 2024; Sadique et al., 2025), comparing pulmonary and hepatic cell models may help elucidate whether MNP-induced responses are organ-specific. Therefore, A549 and HepG2 cells were selected as relevant *in vitro* models, with A549 cells representing pulmonary responses to inhaled particles and HepG2 cells representing hepatic xenobiotic metabolism (Forcella et al., 2022; Kim et al., 2022). In this study, we investigated and compared the genotoxic effects of polystyrene and polyethylene micro- and nanoparticles in HepG2 and A549 cell lines using the alkaline and Fpg-modified comet assays. Comparing these 2 cell models enabled evaluation of MNP-induced cellular responses from distinct tissues, including cytotoxicity, primary DNA damage, ROS generation, and oxidative DNA lesions, and their potential relationships with particle uptake. This study aimed to determine whether these responses follow a common ROS-mediated mechanistic pattern or vary according to polymer type, particle size, and cellular model.

## 2 Materials and methods

### 2.1 Particles and their characterization

All particles were purchased as colloidal suspensions: polystyrene nanoparticles from Spherotech Inc. (United States), and polystyrene microparticles and polyethylene micro- and nanoparticles from Lab261 (United States). According to the manufacturer, the size of the primary polystyrene nanoparticles ranged from 0.05 to 0.1 μm, with an average of 70 nm. Polystyrene microparticles had a primary size of 1.05 μm with a density of 1.05 g/cm^3^. Polyethylene nanoparticles are specified as 0.068 μm in size, with a density of less than 1 g/cm^3^. Polyethylene microparticles are said to be 0.98 μm in size and have a density of less than 1 g/cm^3^. Particle size was assessed by dynamic light scattering (DLS) in complete cell culture medium immediately after sonication and then following 24 h of incubation. The hydrodynamic diameters measured immediately after preparation were 123.1, 1,410, 72.13, and 445.6 nm for PS nanoparticles, PS microparticles, PE nanoparticles, and PE microparticles, respectively, and remained relatively comparable after 24 h, with values of 95.34, 1,195, 74.48, and 445.5 nm, respectively. The corresponding PdI values indicated a relatively low-to-moderate degree of size heterogeneity across the particle dispersions (Supplementary Table S1). Overall, the DLS-derived hydrodynamic diameters were comparable to manufacturer-reported sizes for PS micro- and nanoparticles and for PE nanoparticles, while a substantial difference was observed for PE microparticles.

### 2.2 Cultivation of HepG2 and A549 cells

HepG2 and A549 cells, provided from Merck (cell line no. 85011430 and no. 86012804, respectively, Darmstadt, Germany), were cultured in Dulbecco’s modified Eagle’s medium (BenchStable™ DMEM with GlutaMAX™ supplement, Gibco™, Thermo Fisher Scientific, Waltham, MA, United States) supplemented with 10% fetal bovine serum (FBS, Gibco™) and 1% penicillin/streptomycin solution (PEST, Gibco™). The cells were incubated at 37 °C with 5% CO_2_ as an adherent monolayer. Both cell lines were passaged twice a week when cells reached 70%–80% confluence, using phosphate-buffered saline (PBS, Gibco™) for washing and wet trypsinization with trypsin-EDTA (0.025% Bovine Trypsin and 0.01% EDTA, Gibco™).

### 2.3 Exposures of cells to PS and PE micro- and nanoparticles

Stock suspensions of polystyrene and polyethylene micro- and nanoparticles were prepared in complete cell medium at a concentration of 1 mg/mL (0.1%) for particle uptake and ROS generation analysis, comet assays, and cytotoxicity testing. Prior to use, MNP suspensions were sonicated at 35 kHz for 10 min in a Bandelin Sonorex Super Sonication Bath (BANDELIN electronic GmbH and Co. KG, Berlin, Germany) to break up formed agglomerates and achieve uniform MNP dispersions.

For all experimental procedures, including flow cytometry, cell viability assay, and comet assays analyses, cells were initially seeded into 12-well plates at a density of 4 × 10^4^ cells/mL. The cells were incubated for 24 h at 37 °C in a humidified 5% CO_2_ atmosphere to facilitate cell attachment. Following this initial incubation, the culture medium was replaced with fresh medium containing suspensions of PS and PE micro- and nanoparticles at varying concentrations, depending on the assay. For flow cytometric analysis, cells were treated with 10, 40, 80, and 100 μg/mL of the respective polystyrene micro- or nanoparticles for 24 h. To assess the cytotoxic and genotoxic effects of these particles, cells were exposed to concentrations of 5, 10, 20, 40, 60, 80, and 100 μg/mL for 24 h. The 5–100 μg/mL concentration range was selected based on current recommendations for *in vitro* testing of nanomaterials, which suggest a maximum concentration of 100 μg/mL to minimize excessive particle agglomeration and variability in cellular uptake (Burgum et al., 2024). All exposures were performed at 37 °C in a humidified atmosphere containing 5% CO_2_, after which the cells were subjected to further analysis.

### 2.4 Cell viability

PS and PE micro- and nanoparticle cytotoxicity was determined by cell counts using a dual acridine orange and ethidium bromide staining technique. 20 μL of the cell suspension and 2 µL of acridine orange and ethidium bromide (Carl Roth GmbH + Co. KG, Karlsruhe, Germany) dye mix (∼5 μg/mL of AO and ∼3 μg/mL of EtBr) were mixed, and a cover glass was placed on top, and microscopic analysis was performed (Nikon Eclipse 80i, Nikon, Tokyo, Japan). This dual staining is based on the differential membrane permeability of viable and non-viable cells: acridine orange penetrates intact membranes and stains viable cells green, whereas ethidium bromide enters cells with compromised membranes and stains dead or late-apoptotic cells red/orange due to DNA intercalation (Liu et al., 2015). The cell viability experiments were performed in four independent biological replicates (n = 4). A minimum of 100 cells per treatment was analyzed on each slide to calculate the percentage of dead and viable cells. To avoid false-positive results in the comet assay, it is recommended to keep cytotoxicity below 25% (Azqueta et al., 2022).

### 2.5 Alkaline and Fpg-modified comet assay

The comet assay was conducted under alkaline conditions to detect DNA strand breaks. Additionally, formamidopyrimidine DNA glycosylase (Fpg) (New England Biolabs, Ipswich, MA, United States) was used to assess oxidative DNA lesions, specifically targeting oxidized purine bases, as outlined by Møller et al. (2023). Experiments were conducted in 12-well plates using the same concentrations of polystyrene and polyethylene MNPs as described earlier. Untreated (0 μg/mL) and positive (KBrO_3_, 1.5 mM; Sigma-Aldrich Co., St. Louis, MO, United States) controls were also included. The procedures for conducting the comet assay are outlined in accordance with the recommendations established by MIRCA (Møller et al., 2020). The treated cell suspension was mixed with fresh 1% low-melting-point (LMP; Carl Roth GmbH + Co. KG, Karlsruhe, Germany) agarose at a 3:5 ratio (final LMP concentration 0.625%), pipetted onto slides precoated with 1% normal-melting-point (NMP; Carl Roth GmbH + Co. KG, Karlsruhe, Germany) agarose, and covered with a coverslip. The agarose was allowed to solidify at 4 °C for 10 min. The coverslips were removed, and the slides were immersed in a cold, freshly prepared lysis solution (2.5 M NaCl, 100 mM Na2EDTA, and 10 mM Tris, with 1% Triton X-100 added just prior to use, pH 10; all reagents were purchased from Carl Roth GmbH + Co. KG, Karlsruhe, Germany). The slides were then kept in the dark for 1 h at 4 °C. Following cell lysis, an enzymatic reaction with Fpg was performed. Firstly, the slides were washed three times for 5 min with enzyme reaction buffer (40 mM HEPES, 0.5 mM Na_2_EDTA, 0.1 M KCl, pH 8 (with 10 M KOH); all reagents were purchased from Carl Roth GmbH + Co. KG, Karlsruhe, Germany). A phosphate-buffered saline (PBS) control was included, in which the samples were washed with PBS solution. After washing, the slides were placed on ice and divided into two groups. One group was incubated with 100 μL of enzyme reaction buffer containing bovine serum albumin (BSA, 0.2 mg/mL) (Gibco, United States) and formamidopyrimidine DNA glycosylase (Fpg, 2 U/mL) to detect oxidative DNA lesions. The second group of slides was incubated with 100 μL of enzyme reaction buffer containing 0.2 mg/mL BSA, without Fpg enzyme, to assess single- and double-strand DNA breaks and alkali-labile sites. A cover glass was placed on each slide, and the samples were incubated in a humidified chamber at 37 °C for 30 min. After incubation, the coverslips were removed, and the slides were transferred to a horizontal gel electrophoresis tank (COMET – 20 SYSTEM, Scie-Plas, Cambridge, United Kingdom) filled with cold, fresh electrophoresis buffer (0.3 M NaOH and 1 mM Na_2_EDTA, pH 13). The slides were left in the solution for 20 min to facilitate DNA unwinding, and then electrophoresis was carried out at 20 V and 300 mA (1 V/cm) for 20 min. To maintain a constant buffer temperature, the platform was cooled to 4 °C using a recirculating cooler FL300 (Julabo, Seelbach, Germany), and the buffer was continuously circulated throughout the electrophoresis with a peristaltic pump. After electrophoresis, the slides were neutralized with Tris-HCl buffer (0.4 M Tris-HCl, pH 7.5; Carl Roth GmbH + Co. KG, Karlsruhe, Germany) for 15 min and stained with 60 µL of ethidium bromide (10 μg/mL) (Carl Roth GmbH + Co. KG, Karlsruhe, Germany). To avoid additional light-induced DNA damage, all experimental procedures described above were conducted under dimmed light conditions.

The slides were evaluated blindly by a single scorer using a fluorescence microscope (Nikon Eclipse 80*i*, Japan) at ×400 magnification. Image capture and comet analysis were performed using LUCIA Comet Assay™ software (version 7.60; Laboratory Imaging, s.r.o., Czech Republic). The experiment was performed in four independent biological replicates (n = 4). For each sample, in each replicate, two gels were prepared, and 50 randomly selected nucleoids per gel were analyzed, resulting in a total of 100 comets per sample. The DNA damage was quantified as the percentage of DNA in the comet tail (Tail DNA, %).

### 2.6 Measurement of ROS by flow cytometry

For the analysis of reactive oxygen species (ROS) levels, cells were seeded and exposed to different concentrations of PS and PE micro- and nanoparticles for 24 h, as described in Section 2.3. Following exposure, the cells were detached and centrifuged at 1,000 rpm for 4 min. Untreated cells were used as the negative control, with both stained and unstained controls included. Cells treated with TBHP at a final concentration of 50 μM for 4 h prior to staining were used as the positive control. Subsequently, the cell pellet was resuspended in 25 μM cell-permeable, oxidant-sensitive fluorescent dye, 2,7-dichlorodihydrofluorescein diacetate (H_2_DCFDA) (Abcam, Cambridge, United Kingdom). The samples were incubated for 30 min at 37 °C in the dark and subsequently analyzed by flow cytometry. Approximately 1 × 10^6^ cells from each sample were analyzed. The fluorescence signal of dichlorofluorescein (DCF) was acquired using the FITC channel (excitation: 488 nm; emission: 519 nm). Data analysis was performed with Floreada.io software (https://floreada.io, accessed on 30 March 2025). A gating strategy was applied to exclude debris and cell aggregates prior to the analysis. The main cell population was identified using forward scatter (FSC-A) and side scatter (SSC-A), excluding low-scatter events associated with cellular debris and other non-cellular particles. Doublets and aggregates were excluded using FSC-A versus FSC-H gating, and the resulting singlet population was used for quantitative analysis of DCF fluorescence. The median fluorescence intensity (MFI) was determined and expressed as a fold change relative to the untreated control (0 μg/mL).

### 2.7 Cellular uptake analysis by flow cytometry

The uptake of PS and PE micro- and nanoparticles in HepG2 and A549 cells was assessed using flow cytometry side-scattered light analysis, as described by Suzuki et al. (2007). Following particle exposure as described in Section 2.3, the cell culture medium was aspirated, and the cells were washed with phosphate-buffered saline (PBS). The cells were then wetly trypsinized with trypsin-EDTA solution for 4 min, transferred to sterile 1.5 mL tubes, and centrifuged at 1,000 rpm for 4 min. The cell pellet was resuspended in 1 mL of PBS. Cell samples were analyzed using a FACSCalibur™ flow cytometer (BD Biosciences, Germany). Approximately 1 × 10^6^ cells from each sample were analyzed, and the data were processed using the floreada.io software. The singlet population was obtained as described in 2.6. Forward scatter (FSC-A) light, representing cell size, and side scatter (SSC-A) light, reflecting intracellular complexity and granularity, were measured, and changes in their signal intensities were visualized as histograms as an indirect assessment of PS and PE micro- and nanoparticle uptake.

### 2.8 Statistics and data presentation

Statistical analyses and graph preparation were performed using GraphPad Prism version 10.3.1 (464) (GraphPad Software, San Diego, United States). Cell viability data were analyzed using one-way analysis of variance (ANOVA) with the Geisser-Greenhouse correction, followed by Dunnett’s multiple comparisons test to compare each treatment group with the untreated control. For ROS measurements, fold-change values from three independent experiments were calculated relative to the untreated control. For each treatment condition, a two-tailed one-sample t-test was performed to assess whether the mean fold-change differed from the expected value of 1. A p-value <0.05 was considered statistically significant. For the comet assay data, normality was first assessed using the Shapiro-Wilk test. The mean Tail DNA (%) value was calculated for each independent replicate, based on 100 comets (50 comets per gel from two gels). These values were used as the statistical unit for analyses, with the four independent experiments serving as biological replicates. As the assumption of normality was met, differences in DNA damage among polystyrene and polyethylene micro- and nanoplastic concentration groups were evaluated using ordinary one-way ANOVA followed by Dunnett’s multiple comparisons test. Statistical significance was established at *p* < 0.05. In addition to comparisons with the untreated control, DNA damage responses were compared across polymer type (PS vs. PE), particle size (nano vs. micro), and cell line (A549 vs. HepG2). For the alkaline comet assay, treatment-related DNA damage was expressed as a relative to the matched untreated control. A linear mixed-effects model was fitted with polymer, particle size, cell line, and concentration as fixed effects, including polymer × size × cell line interactions, and biological replicate as a random intercept. For the Fpg-modified comet assay, Fpg-sensitive sites were calculated as the difference between Fpg- and enzyme buffer-treated samples and expressed relative to the matched untreated control. As no clear linear concentration-response relationship was observed, concentration was treated as a categorical factor. The same mixed-effects framework was applied, with interactions among polymer, particle size, and cell line evaluated. For significant interactions, estimated marginal means and Holm-adjusted pairwise comparisons were performed. Degrees of freedom were estimated using the Satterthwaite approximation, and p < 0.05 was considered statistically significant. Analyses were performed using the GAMLj module in jamovi version 28.2.

## 3 Results

### 3.1 Cell viability

Cell viability following 24-h exposure to polystyrene and polyethylene particles (0–100 μg/mL) was evaluated using acridine orange/ethidium bromide dual staining. Overall, exposure to all tested particles resulted in only a slight reduction in viability, approximately 10%, indicating no pronounced cytotoxic effects (Figure 1).

**FIGURE 1:**
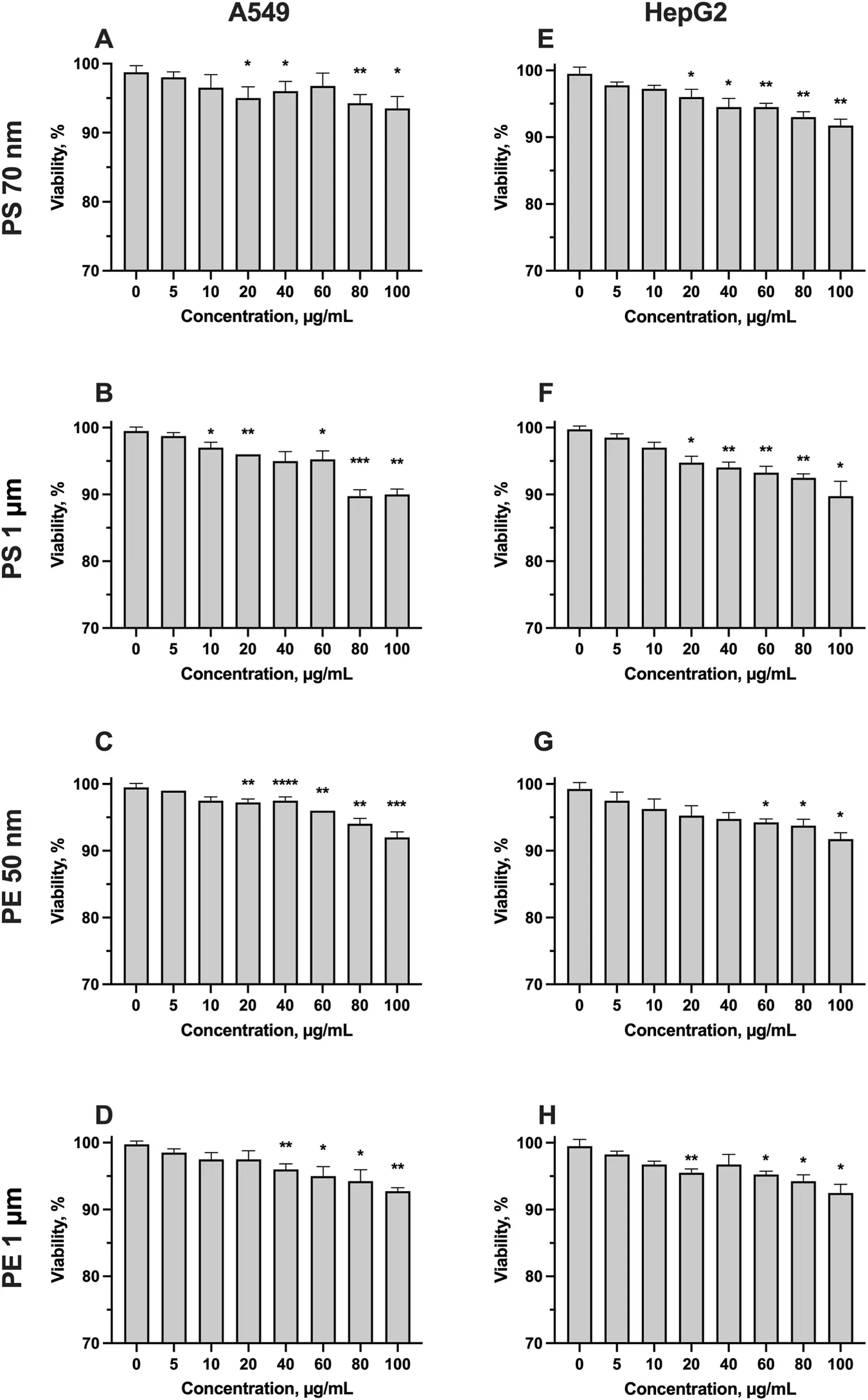
Percentage of viable cells after 24-h exposure of A549 **(A–D)** and HepG2 **(E–H)** cells to different sizes of PS and PE particle concentrations (5–100 μg/mL). Results are shown as mean ± SD (n = 4). A statistically significant decrease (p < 0.05) compared to the untreated control group is indicated by an asterisk (*p < 0.05, **p < 0.01, ***p < 0.001, ****p < 0.0001).

In the A549 cell line, exposure to PS micro- and nanoparticles caused a gradual, concentration-dependent decrease in viability. Statistically significant (*p* < 0.05) reductions compared with the untreated control were observed at 20, 40, 80, and 100 μg/mL when cells were treated with polystyrene 70 nm nanoparticles (Figure 1A). A similar but more pronounced trend was observed following exposure to PS microparticles (1 µm) (Figure 1B), with a significant decrease in viability from 10 μg/mL onward, reaching the strongest effect at 80 μg/mL. Polyethylene nanoparticles (Figure 1C) also induced a concentration-dependent decline in viability. Significant differences relative to the untreated control were observed from 20 to 100 μg/mL, with 40 μg/mL showing the most pronounced response. PE microparticles (Figure 1D) produced a similar trend, but the level of statistical significance was lower.

In the HepG2 cell line, all four particle types similarly produced only minor reductions in viability. Exposure to both PS micro- and nanoparticles (Figures 1E,F, respectively) resulted in similar concentration-dependent reductions in cell viability. Statistically significant decreases compared with the untreated control were observed at all concentrations except the two lowest. Compared with PS particles, PE nanoparticles (Figure 1G) exerted a less pronounced effect on cell viability, with statistically significant (*p* < 0.05) reductions observed only at 60, 80, and 100 μg/mL. Similarly, PE microparticles (Figure 1H) produced relatively modest but statistically significant decreases in cell viability at 20, 60, 80, and 100 μg/mL.

Overall, both cell lines exhibited concentration-dependent reduction in viability following exposure to PS and PE micro and nanoparticles. A549 cells appeared to be more sensitive to PE particles, particularly 50 nm nanoparticles, which induced a significant decrease across a broader concentration range and with greater statistical significance. In contrast, HepG2 cells showed a more pronounced response to PS particles, with both nano and microparticles causing significant reductions at nearly all concentrations above the lowest tested. Despite the observed differences, cell viability remained above the recommended cytotoxicity threshold in both cell lines, indicating that neither PS nor PE particles induced cytotoxicity could confound the interpretation of the genotoxicity results (Azqueta et al., 2022).

### 3.2 Primary DNA damage induced by PS and PE micro- and nanoparticles

Primary DNA damage was assessed using the alkaline comet assay following 24 h of exposure of cell lines A549 and HepG2 to PS and PE micro- and nanoparticles at concentrations ranging from 5 to 100 μg/mL. The extent of DNA damage was greater in HepG2 cells than in A549 cells, although the response varied with particle type and size (Figure 2).

**FIGURE 2:**
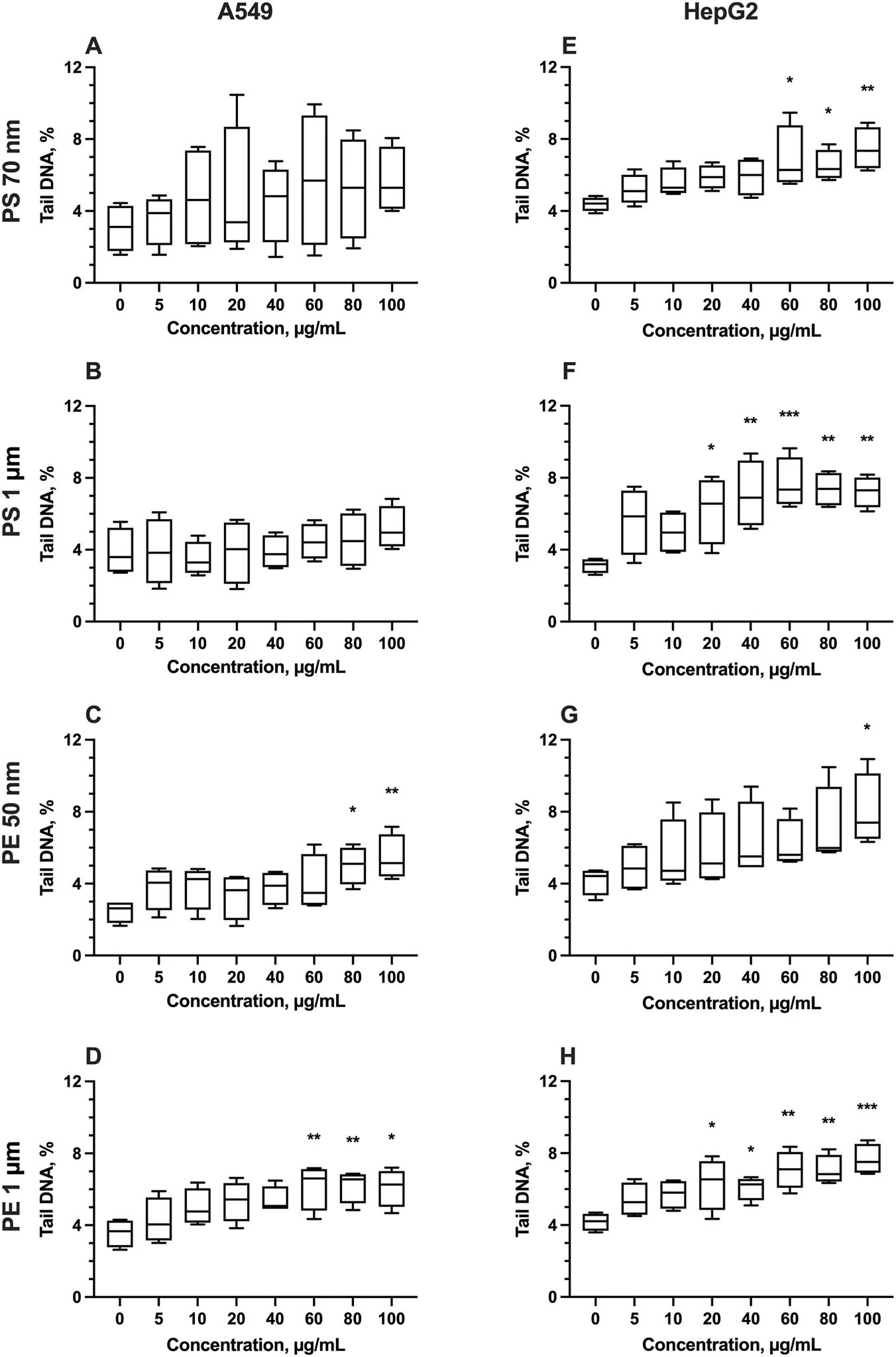
Percentage of primary DNA in a comet tail after 24-h exposure of A549 **(A–D)** and HepG2 **(E–H)** cells to different sizes of PS and PE particle concentrations (5–100 μg/mL). Results are presented as box plots representing four independent biological replicates. The median is indicated by a horizontal line within the box, the interquartile range is depicted by the box, and the whiskers show the minimum and maximum values. Statistical analysis was performed using one-way ANOVA, followed by Dunnett’s multiple comparisons test. Statistically significant changes (*p < 0.05, **p < 0.01, ***p < 0.001) compared to the untreated control group are indicated by an asterisk.

In A549 cells, exposure to PS 70 nm nanoparticles (Figure 2A) resulted in a slight concentration-dependent increase in tail DNA (%). Although DNA damage tended to increase at higher concentrations, none of the tested concentrations differed significantly from the untreated control. Similarly, PS 1 µm microparticles (Figure 2B) produced only minor increases in tail DNA (%), and no statistically significant differences were observed throughout the tested concentration range. In contrast, PE nanoparticles (Figure 2C) induced a concentration-dependent increase in primary DNA damage. Statistically significant increases in tail DNA (%) compared with the control were observed at the highest concentrations – 80 μg/mL and 100 μg/mL. A similar response was observed following exposure to PE microparticles (Figure 2D), with significantly elevated DNA damage at 60, 80, and 100 μg/mL.

HepG2 exhibited greater apparent sensitivity to polystyrene particle exposure than the A549 cell line (p < 0.001). Exposure to PS nanoparticles (Figure 2E) resulted in a gradual increase in tail DNA (%), reaching statistical significance at 60 μg/mL, 80 μg/mL, and 100 μg/mL. An even more pronounced response was observed following exposure to PS microparticles (Figure 2F), which significantly increased DNA damage at almost all concentrations, reaching the highest level at 60 μg/mL. Exposure to polyethylene nanoparticles (Figure 2G) resulted in a modest increase in DNA damage, with a statistically significant effect observed only at the highest concentration tested (100 μg/mL, p < 0.05). In contrast, PE microparticles (Figure 2H) caused a clear, concentration-dependent increase in tail DNA percentage, with statistically significant elevations noted at 20, 40, 60, 80, and 100 μg/mL.

Comparative analysis using a linear mixed-effects model demonstrated that primary DNA damage was influenced by particle size (p = 0.004), cell line (p < 0.001), and concentration (p < 0.001), whereas no significant overall effect of polymer type was observed. Significant polymer × cell-line, size x cell-line, and polymer x size x cell-line interactions were detected (all p < 0.001), indicating that the effects of polymer composition and particle size differed between A549 and HepG2 cells. The strongest response was observed for PS microparticles in HepG2 cells, which showed significantly greater DNA damage than PS microparticles in A549 cells and PS nanoparticles in HepG2 cells (both p < 0.001). Furthermore, PE induced greater DNA damage than PS in A549 cells, whereas the opposite pattern was observed in HepG2 cells (both p < 0.001).

### 3.3 ROS induction

To determine whether the observed cytotoxic and genotoxic responses were associated with ROS-mediated mechanisms, intracellular reactive oxygen species generation was assessed using the H_2_DCFDA fluorescent probe in conjunction with flow cytometry. Cells were exposed to PS and PE micro- and nanoparticles at concentrations of 10, 40, 80, and 100 μg/mL for 24 h. DCF fluorescence distributions were evaluated together with median fluorescence intensity (MFI), and MFI values were expressed as fold-change relative to the untreated control (Figure 3). The fold-change values, standard deviations, and corresponding *p*-values for all tested particles and concentrations are provided in Supplementary Table S2.

**FIGURE 3:**
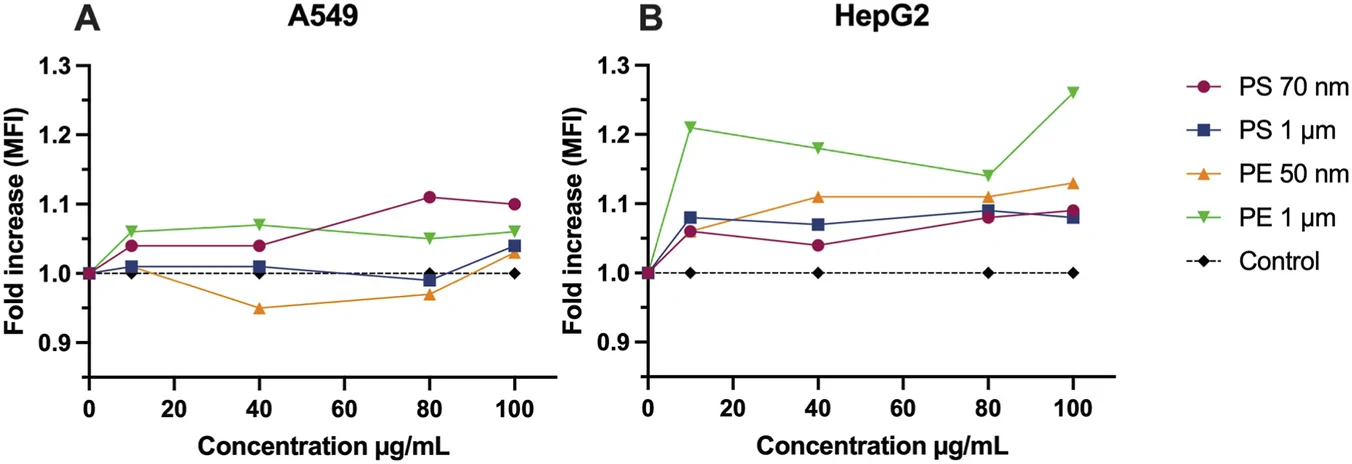
Changes in ROS-associated DCF fluorescence in A549 **(A)** and HepG2 **(B)** cells exposed to polystyrene (PS) and polyethylene (PE) micro- and nanoparticles. DCF fluorescence was quantified as median fluorescence intensity (MFI) and expressed as an average fold-change relative to the untreated control (0 μg/mL) from three independent biological replicates.

In A549 cells (Figure 3A), exposure to polystyrene and polyethylene micro- and nanoparticles resulted in ROS levels generally comparable to those of the untreated control, with no apparent changes observed following exposure to PS 1 µm and PE 50 nm particles. A slight increase in ROS levels was observed following exposure to PS 70 nm and PE 1 µm particles; however, these changes were not statistically significant. In HepG2 cells (Figure 3B), ROS levels also remained comparable to those of the untreated control across the tested PS and PE particles and concentrations. Exposure to PS 70 nm nanoparticles resulted in slight but statistically significant increases in ROS levels at 10 and 40 μg/mL (1.06-fold, p=0.049; 1.04-fold, p=0.032 , respectively). PS 1 µm particles produced slight increases in ROS levels, although none of the changes were statistically significant. For PE 50 nm nanoparticles, a slight but statistically significant increase was observed at 40 μg/mL (1.11-fold, p=0.024 ), while the changes at the other concentrations were not statistically significant. PE 1 µm particles showed more pronounced numerical increase in ROS levels, ranging from 1.14- to 1.26-fold, with the highest increase observed at 100 μg/mL; however, none of these changes reached statistical significance.

No consistent concentration-dependent increase in ROS levels was observed in either cell model. The most pronounced changes were observed in HepG2 cells, whereas ROS levels in A549 cells remained generally comparable to those of the untreated control across the tested exposure conditions.

### 3.4 Oxidative DNA damage induced by PS and PE micro- and nanoparticles

To assess whether the observed subtle ROS responses were associated with oxidative DNA damage, the Fpg-modified comet assay was performed after 24 h of exposure of the A549 and HepG2 cell lines to polystyrene and polyethylene micro- and nanoparticles at concentrations of 5–100 μg/mL (Figure 4).

**FIGURE 4:**
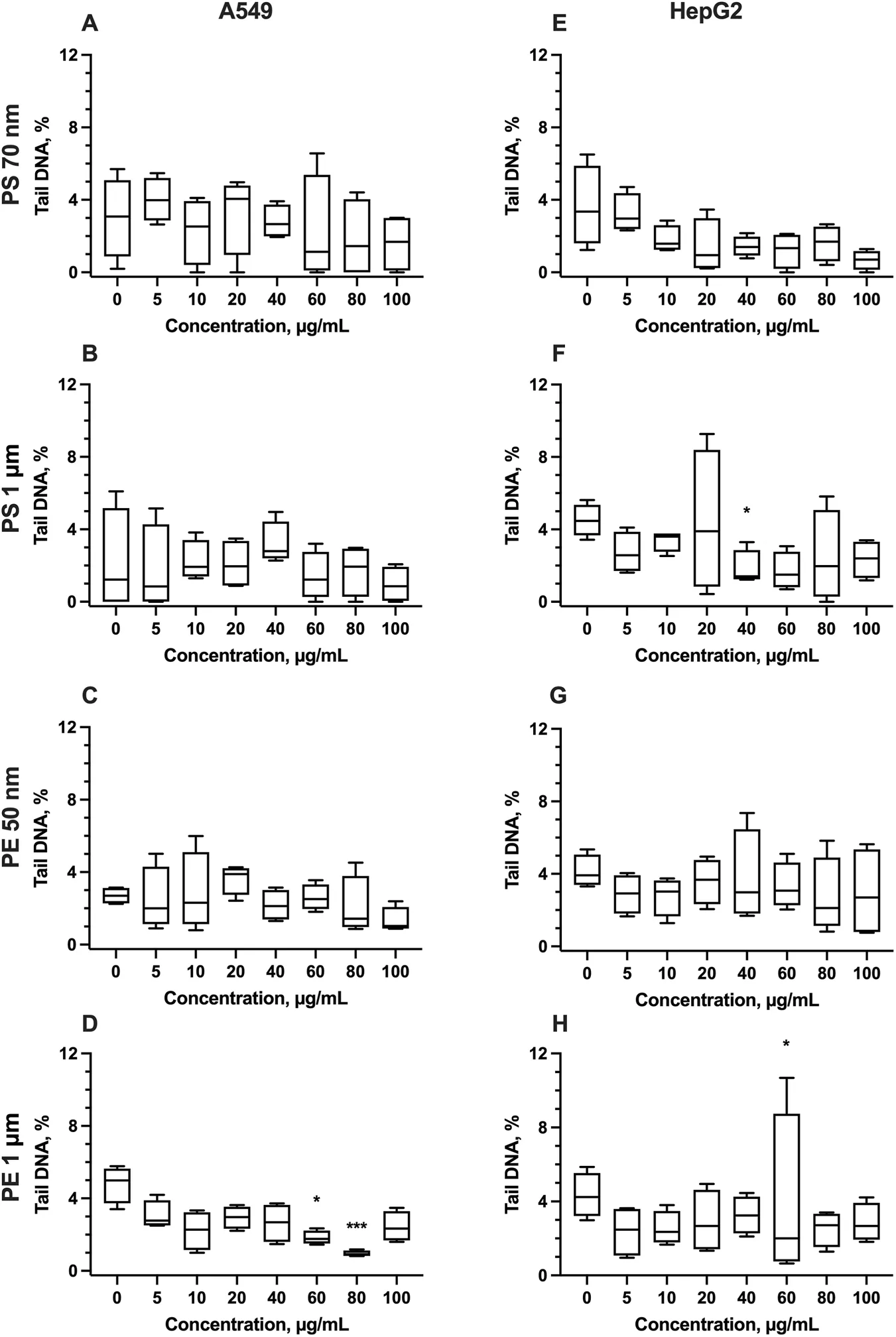
Percentage of oxidative DNA in a comet tail after 24-h exposure of A549 **(A–D)** and HepG2 **(E–H)** cells to different sizes of PS and PE particle concentrations (5–100 μg/mL). Results are presented as box plots representing four independent biological replicates. The median is indicated by a horizontal line within the box, the interquartile range is depicted by the box, and the whiskers show the minimum and maximum values. Statistical analysis was performed using one-way ANOVA, followed by Dunnett’s multiple comparisons test. Statistically significant changes (*p < 0.05, ***p < 0.001) compared to the untreated control group are indicated by an asterisk.

In A549 cells, exposure to 70 nm PS nanoparticles (Figure 4A) resulted in slightly elevated levels of oxidative DNA damage at concentrations of 5 and 20 μg/mL compared to the untreated control, as indicated by an increase in Tail DNA (%). However, these differences were not statistically significant. Similarly, exposure to PS microparticles (Figure 4B) produced slightly higher Tail DNA percentage than the control at 10, 20, 40 and 80 μg/mL, although none of these differences reached statistical significance. Likewise, PE micro- and nanoparticles (Figures 4C,D) did not show an increase in oxidative DNA damage at any tested concentration, with Tail DNA (%) remaining comparable to or below the control level.

HepG2 cells exposed to PS micro- and nanoparticles (Figures 4E,F) did not exhibit oxidative DNA damage across the tested concentration ranges. The percentage of tail DNA generally remained similar to or lower than that of the untreated control, with no observable concentration-dependent response. Similarly, exposure to PE particles, including those of 50 nm and 1 µm (see Figures 4G,H), did not induce oxidative DNA damage. Although tail DNA showed variable responses across concentrations, overall, neither PS nor PE particles increased oxidative DNA damage in HepG2 cells.

The comparative analyses of Fpg-sensitive sites did not reveal evidence of a consistent concentration-dependent increase in oxidative DNA damage. When concentration was treated as a categorical factor, no significant main effects of polymer type, particle size, cell line, or concentration were observed; none of the interactions involving concentration were significant. Significant polymer x size (p = 0.002) and polymer x cell line (p < 0.001) interactions were detected, whereas size x cell line and polymer x size x cell line interactions were not significant. Holm-adjusted *post hoc* comparisons identified differences between PE micro- and nanoparticles (p = 0.003), between PE and PS microparticles (p = 0.029), between PE and PS in A549 cells (p = 0.017), and between A549 and HepG2 cells following PS exposure (p = 0.002). However, the corresponding model-adjusted values were close to zero or negative in untreated controls, indicating that these differences reflected variation in response to stimulus magnitude rather than a consistent induction of oxidative DNA lesions.

### 3.5 Cellular uptake analysis

To further investigate whether differences in cellular responses are related to particle internalization, the uptake of micro- and nanoplastic particles by HepG2 and A549 cells was assessed using flow cytometric side-scatter light analysis (Figure 5). The cells were exposed to polystyrene and polyethylene particles ranging from 0 to 100 μg/mL for 24 h, and side-scatter light (SSC-A) intensities were measured to assess potential MNP uptake.

**FIGURE 5:**
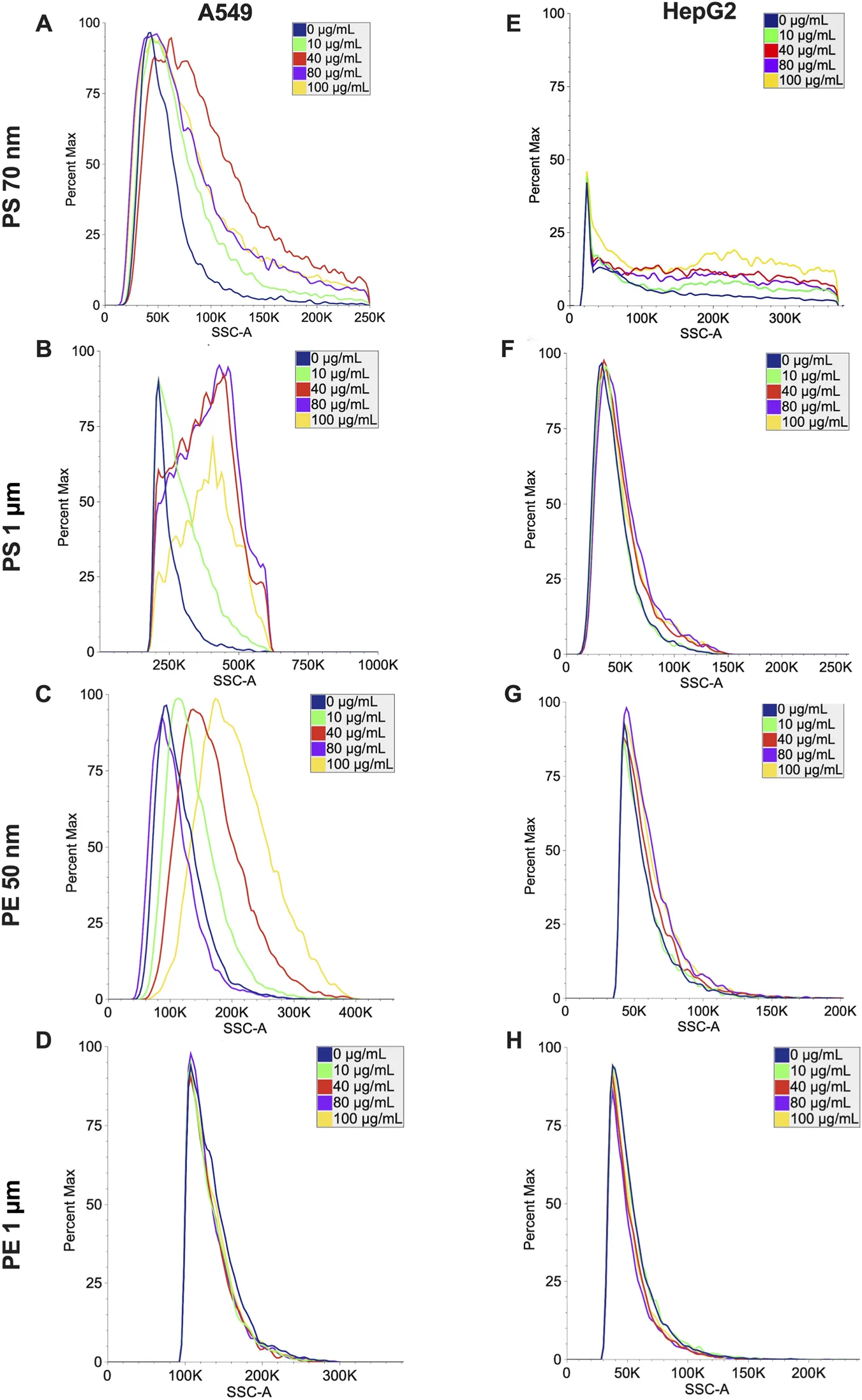
Analysis of different polymer and size plastic particles uptake by A549 **(A–D)** and HepG2 **(E–H)** cells using flow cytometry. Both cell lines were treated with different concentrations of PS and PE micro- and nanoparticles (10, 40, 80, and 100 μg/mL) for 24‐h. Observed changes in side-scattered light intensity (SSC‐A) were analyzed as an indicator of intracellular complexity.

In A549 cells (Figures 5A–D), exposure to 70 nm polystyrene (PS) nanoparticles (Figure 5A) caused a rightward shift in the side scatter (SSC) histograms, indicating that there was increased intracellular complexity following particle exposure. The SSC shift generally increased with higher particle concentrations, although the response was not strictly linear at the highest concentrations. In contrast, 1 μm PS microparticles (Figure 5B) produced a distinct SSC profile, with the most pronounced changes observed at intermediate concentrations (40 and 80 μg/mL). Exposure to polyethylene (PE) micro- and nanoparticles (Figures 5C,D) resulted in concentration-dependent SSC shift caused by 50 nm PE nanoparticles, while, in contrast, 1 μm PE microparticles produced only minimal changes in SSC distribution.

HepG2 cells (Figures 5E–H) also exhibited increased SSC following particle exposure, although the magnitude of the response was generally lower than that observed in A549 cells. Both 70 nm PS and 50 nm PE nanoparticles (Figures 5E,G) caused modest rightward shifts in the SSC histograms, with most distributions remaining close to the untreated control. Similarly, exposure to 1 μm PS and PE microparticles (Figures 5F,H) resulted in only minor alterations across tested concentrations compared to untreated controls. These findings suggest that HepG2 cells exhibit a lower SSC response than A549 cells.

Overall, PS particles elicited greater SSC changes than PE particles in both A549 and HepG2 cells, with the effect being more pronounced in A549 cells, suggesting greater alterations in intracellular complexity.

## 4 Discussion

This study investigated the cytotoxic and genotoxic effects of PS and PE micro- and nanoparticles in A549 and HepG2 cell lines. Cells were exposed to a range of particle concentrations (5–100 μg/mL) for 24 h, after which cell viability was assessed by dual acridine orange and ethidium bromide staining, and primary and oxidative DNA damage were evaluated using the alkaline and Fpg-modified comet assay, respectively. Cellular uptake of the particles and ROS generation were evaluated using flow cytometry. The results are summarized in Supplementary Table S3.

In the present study, exposure to PS and PE particles resulted in a significant but minor decrease in cell viability in both A549 and HepG2 cells, indicating that the tested particles did not exert strong cytotoxic effects under the tested concentrations. Nevertheless, cell line-specific differences were observed, with A549 cells showing greater sensitivity to PE particles, particularly 50 nm nanoparticles, whereas HepG2 cells exhibited a more pronounced decrease in viability after exposure to both PS micro- and nanoparticles. These findings are generally consistent with previous studies reporting limited cytotoxicity of micro- and nanoplastics in A549 cells. The cytotoxicity showed a dose-dependent response, but it remained relatively low even at the highest PS concentrations tested (Babonaitė et al., 2023; Koner and Ramasubbu, 2026). Likewise, PET microplastics have been reported to cause only a slight (Brito et al., 2024) or no decrease (Milillo et al., 2024) in A549 cell viability, while PE microparticles induced only a slight decrease at the highest tested concentrations (Gautam et al., 2022). Similarly, exposure to PS microparticles did not reduce A549 cell viability below 95%, even after prolonged exposure (Goodman et al., 2021). Comparable observations have also been reported for HepG2 cells. PS nanoparticles reduced cell viability in a concentration-dependent manner, although cell viability remained above 75%–80% even at the highest concentrations tested (He et al., 2020; Guo et al., 2024). In contrast, PE microparticles exhibited little or no cytotoxicity in HepG2 cells, with viability generally remaining above 80%–90% (Stock et al., 2021; Najahi et al., 2025), while exposure to PS microparticles maintained cell viability above 94% (Goodman et al., 2022). These findings support the present study’s results, indicating that although PS and PE particles may induce modest, cell-type-dependent reductions in viability, they are generally not markedly cytotoxic under the exposure conditions.

Given the limited cytotoxicity observed under the tested conditions, the primary DNA damage was further evaluated to assess the genotoxic potential of the tested particles. Comparative analysis showed that primary DNA damage was significantly influenced by particle size, cell line, and concentration. A more pronounced primary DNA damage response was observed in HepG2 cells than in A549 cells, particularly following exposure to PS microparticles. In contrast, PE induced greater DNA damage than PS in A549 cells, whereas an opposite pattern was observed in HepG2 cells. Previous studies investigating PS-induced genotoxicity in A549 cells have reported inconsistent findings. Liu et al. (2015) observed no increase in DNA strand breaks following exposure to aminated PS nanoparticles, whereas Ernhofer et al. (2025) reported increased DNA damage after exposure to PS nanoparticles. Similarly, Laganà et al. (2023) found that neither nano nor micro-sized PS particles induced significant DNA damage in A549 cells, whereas Babonaitė et al. (2023) reported a significant increase in primary DNA damage in PBMCs following exposure to PS nanoparticles. These discrepancies may be explained by differences in particle surface chemistry (Liu et al. investigated aminated PS nanoparticles rather than non-functionalized PS particles) and by variations in particle size, exposure conditions, cell types, and experimental design. Furthermore, studies using PET particles have demonstrated increased DNA strand breaks in A549 cells (Alzaben et al., 2023), showing that the genotoxic potential of plastic particles may also depend on polymer type. A greater susceptibility of HepG2 cells observed in the present study is supported by previous investigations demonstrating concentration-dependent increases in DNA strand breaks following exposure to PET particles (Roursgaard et al., 2022), with similar dose-dependent responses also reported in other studies employing high PET concentrations. In contrast, no significant DNA damage was detected following acute (24 h) PET exposure in BEAS-2B cells (Gutiérrez-García et al., 2025), while PS nanoparticles failed to induce significant DNA damage in TK6 and THP-1 cells (Rubio et al., 2020). Collectively, these findings suggest that cellular susceptibility to plastic-induced genotoxicity is not determined solely by polymer type but may vary with particle size and cellular model, with the relative contributions of these factors differing across cell lines.

Intracellular ROS generation was evaluated to determine whether oxidative stress could contribute to the observed genotoxic effects following exposure to PS and PE particles. In the present study, the observed changes in DCF fluorescence were generally modest, and no consistent concentration-dependent increase in ROS levels was observed in either A549 or HepG2 cells. In A549 cells, ROS levels remained comparable to those of the untreated control across the tested PS and PE particles and concentrations, whereas in HepG2 cells, statistically significant increases were observed at selected concentrations following exposure to 70 nm PS and 50 nm PE nanoparticles, although these changes were small and did not follow a consistent concentration-dependent pattern. Previous studies investigating the effects of plastic particles on HepG2 cells have reported variable findings. While PS nanoparticles did not significantly increase ROS generation, PET and PVC nanoparticles induced significant ROS production at higher concentrations (100 μg/mL) (Ma et al., 2024). Similarly, another study demonstrated that exposure to PE and PET microparticles significantly increased intracellular ROS levels, confirming that microplastics can induce oxidative stress in hepatic cells (Najahi et al., 2025). The observed discrepancies may be attributed to differences in polymer type and particle size, highlighting that plastic particle characteristics strongly influence ROS induction. In A549 cells, previous studies have also reported particle-dependent effects on ROS generation. Shi et al. (2022) demonstrated that PS nanoparticles induced a concentration-dependent increase in ROS production, whereas PS microparticles produced a weaker response. Xu et al. (2019) likewise reported increased intracellular ROS levels following 24 h exposure to PS microparticles. In contrast, Brito et al. (2024) observed no significant increase in ROS levels in A549 cells exposed to PET microparticles of a size comparable to the PE microparticles investigated in the present study. Together with the present findings, these observations suggest that ROS generation is not determined solely by particle size but may also depend on polymer composition and other physicochemical characteristics.

To further evaluate whether the observed slight ROS responses were linked to oxidative DNA damage, oxidative DNA lesions were evaluated using the Fpg-modified comet assay. Comparative analysis revealed no significant effects of polymer type, particle size, cell line, or concentration. Overall, the absence of a concentration-dependent Fpg response was consistent with the minimal ROS responses observed in the present study, although the available ROS data do not allow a definitive mechanistic relationship to be established. This observation differs from the findings of Malinowska et al. (2023), who reported significant oxidative DNA damage following exposure of peripheral blood mononuclear cells (PBMCs) to PS nanoparticles, as measured by the Fpg-modified comet assay. Similarly, Rubio et al. (2020) reported oxidative DNA damage in human lymphoblastic TK-6 and Raji B cells following exposure to PS nanoparticles, with oxidative DNA damage detected in TK-6 cells across all tested concentrations (5–50 μg/mL) and in Raji B cells only at 50 μg/mL. The discrepancy between these findings and the present results may be attributed to differences in the cellular models, particle characteristics, exposure conditions, and DNA repair capacities. In particular, PBMCs and lymphoblastic cells may respond differently to particle exposure than epithelial-derived A549 and HepG2 cells. These findings suggest that, under the present exposure conditions, the observed primary DNA damage was not accompanied by a consistent increase in Fpg-sensitive oxidative DNA lesions.

To further explore whether differences in particle internalization could contribute to the distinct cellular responses observed between A549 and HepG2 cells, particle uptake was assessed. The observed increase in SSC signal following the exposure to PS and PE particles supports particle-associated alterations in intracellular complexity, consistent with previous studies demonstrating that micro- and nanoplastics are readily taken up by A549 and that the uptake is influenced by particle size, nanosized particles being generally taken up more rapidly than the larger ones (Xu et al., 2019; Liu et al., 2021). Consistent with our findings, previous studies have also confirmed efficient uptake of PS nanoparticles by HepG2 cells, demonstrating that 50–70 nm particles accumulate intracellularly (He et al., 2020). In addition to nanoparticle uptake, studies demonstrate that HepG2 cells can internalize micro-sized PS particles, with prolonged exposure resulting in increased intracellular particle accumulation (Goodman et al., 2022). Efficient cellular uptake has also been described for PE particles. Electron microscopy studies have shown that PE particles (1–2 µm) are internalized by A549 cells (Bengalli et al., 2022). Although intracellular localization was not assessed in the present study, the more pronounced SSC signal observed in A549 cells following PE exposure is consistent with particle-associated changes in side-scattered light intensity. In contrast to A549 cells, only a slight increase in SSC signal was observed in HepG2 cells following PE exposure. This finding is aligned with previous studies reporting limited intracellular uptake of PE microparticles by HepG2 cells, in which only a small proportion of the administered particles were detected intracellularly after exposure (Stock et al., 2021; Ma et al., 2024).

Overall, the relationship between cytotoxicity, genotoxicity, ROS generation, and uptake differed between the pulmonary and hepatic cell models. Primary DNA damage was not accompanied by consistent evidence of oxidative DNA damage or marked ROS generation under the experimental conditions tested, providing no clear support for oxidative base damage as the predominant mechanism underlying the observed genotoxic responses. However, other oxidative or non-oxidative mechanisms may contribute and require further investigation.

The present study contributes to the understanding of PE toxicity, which has received less attention than PS in previous studies, and provides additional evidence on DNA damage induced by micro- and nanoplastics. Moreover, studies evaluating oxidative DNA damage induced by micro- and nanoplastics using the Fpg-modified comet assay remain scarce, underscoring the novelty of this work. By simultaneously evaluating viability, primary and oxidative DNA damage, ROS generation, and particle uptake in 2 cell models, this study provides an integrated assessment of potential cellular responses to PS and PE particles. Nevertheless, the findings should be interpreted considering the limitations of 2D cell cultures and acute exposure conditions, as well as the use of immortalized cell lines, which may not fully reflect the physiological functions of normal tissue. Additionally, the selected concentration range based on current *in vitro* testing recommendations may not capture the complexity and variability of human environmental exposure. An additional limitation concerns the assessment of intracellular ROS generation. Given that H_2_DCFDA serves as a general marker of oxidative stress but lacks sensitivity toward certain primary ROS fractions, the observed changes in DCF fluorescence primarily reflect overall shifts in the cellular redox state rather than specific radical accumulation.

Future studies should further investigate the mechanisms underlying the effects of PS and PE micro- and nanoparticles. Additional quantitative measurements using species-specific probes, alongside detailed assessments of downstream oxidative damage, would be beneficial for fully elucidating the cellular impact observed in the present study. Further research should also consider chronic or repeated low-dose exposure models to better reflect prolonged environmental exposure to micro- and nanoplastics. In addition, investigating both oxidative and non-oxidative mechanisms of DNA damage would help provide a more comprehensive understanding of the genotoxic effects induced by these particles. Studies that combine intracellular localization approaches with genotoxicity endpoints could further clarify whether differences in particle–cell association contribute to the distinct responses observed across cell models.

## Data Availability

The raw data supporting the conclusions of this article will be made available by the authors, without undue reservation.
