# Supplementary material for: Genotoxicity of polystyrene and polyethylene micro- and nanoparticles in human lung (A549) and liver (HepG2) cell lines
Source: Front Toxicol. 2026 Oct 21;8:1962775. doi: 10.3389/ftox.2026.1962775 (PMC13730466; doi:10.3389/ftox.2026.1962775)
Supplement: Supplementary file 1 [file DataSheet1.pdf]

Supplementary Material

Plastic particles	Incubation time (h)	Average (nm)	PdI
**PS 70 nm**	0	123.1	0.184
	24	95.34	0.164
**PS 1 um**	0	1410	0.232
	24	1195	0.314
**PE 50 nm**	0	72.13	0.294
	24	74.48	0.298
**PE 1 um**	0	445.6	0.162
	24	445.5	0.204

Supplementary Table 1. Hydrodynamic particle size and polydispersity index (PdI) determined by dynamic light scattering (DLS) before and after 24 hours of incubation in complete cell culture medium. PS – polystyrene, PE – polyethylene.
