# Supplementary material for: Genotoxicity of polystyrene and polyethylene micro- and nanoparticles in human lung (A549) and liver (HepG2) cell lines
Source: Front Toxicol. 2026 Oct 21;8:1962775. doi: 10.3389/ftox.2026.1962775 (PMC13730466; doi:10.3389/ftox.2026.1962775)
Supplement: Supplementary file 2 [file DataSheet2.pdf]

Supplementary Material

A549				
Micro- and nanoplastics	Concentration µg/mL	Fold-change	SD	*p*-value
PS 70 nm	10	1.04	0.08	0.549
	40	1.04	0.09	0.565
	80	1.11	0.13	0.283
	100	1.10	0.15	0.337
PS 1 µm	10	1.01	0.03	0.456
	40	1.01	0.01	0.225
	80	0.99	0.02	0.529
	100	1.04	0.07	0.377
PE 50 nm	10	1.01	0.12	0.933
	40	0.95	0.12	0.508
	80	0.97	0.12	0.696
	100	1.03	0.11	0.687
PE 1 µm	10	1.06	0.10	0.461
	40	1.07	0.09	0.332
	80	1.05	0.06	0.336
	100	1.06	0.06	0.241
**HepG2**				
Micro- and nanoplastics	Concentration µg/mL	Fold-change	SD	*p*-value
PS 70 nm	10	1.06	0.03	**0.049**
	40	1.04	0.01	**0.032**
	80	1.08	0.07	0.178
	100	1.09	0.08	0.204
PS 1 µm	10	1.08	0.04	0.074
	40	1.07	0.10	0.365
	80	1.09	0.06	0.130
	100	1.08	0.10	0.288

PE 50 nm	10	1.06	0.06	0.238
	40	1.11	0.03	**0.024**
	80	1.11	0.08	0.138
	100	1.13	0.09	0.137
PE 1 µm	10	1.21	0.14	0.115
	40	1.18	0.12	0.127
	80	1.14	0.14	0.220
	100	1.26	0.19	0.144

Supplementary Table 2. Mean fold-change in reactive oxygen species (ROS) levels and statistical analysis in A549 and HepG2 cells following micro- and nanoplastic exposure. PS – polystyrene, PE – polyethylene, SD – standard deviation.
