# Supplementary material for: Genotoxicity of polystyrene and polyethylene micro- and nanoparticles in human lung (A549) and liver (HepG2) cell lines
Source: Front Toxicol. 2026 Oct 21;8:1962775. doi: 10.3389/ftox.2026.1962775 (PMC13730466; doi:10.3389/ftox.2026.1962775)
Supplement: Supplementary file 3 [file DataSheet3.pdf]

Supplementary Material

Plastic particles	Cell Line	Viability	Primary DNA damage	ROS response	Oxidative DNA damage	Uptake (SSC signal)
PS 70 nm	A549	Slight C-D decrease	No significant increase	Slight increase	No significant increase	Pronounced shift
	HepG2	Slight C-D decrease	Significant at 60, 80 and 100 µg/mL	Slight, significant increase	No increase	Slight shift
PS 1 µm	A549	Slight C-D decrease	No significant increase	No apparent change	No significant increase	Pronounced shift
	HepG2	Slight C-D decrease	Significant C-D increase	Slight increase	No increase	Slight shift
PE 50 nm	A549	Slight C-D decrease	Significant at 80 and 100 µg/mL	No apparent change	No significant increase	Pronounced shift
	HepG2	Slight C-D decrease	Significant at 100 µg/mL	Slight, significant increase	No increase	Slight shift
PE 1 µm	A549	Slight C-D decrease	Significant at 60, 80 and 100 µg/mL	Slight increase	No increase	Slight shift
	HepG2	Slight C-D decrease	Significant C-D increase	Modest, not significant increase	No increase	Slight shift

Supplementary Table 3. Summary of cellular responses to PS and PE particles in A549 and HepG2 cells. PS – polystyrene, PE – polyethylene, C-D – concentration-dependent, ROS – reactive oxygen species, SSC – side scattering light.
